# LASP1 promotes nasopharyngeal carcinoma progression through negatively regulation of the tumor suppressor PTEN

**DOI:** 10.1038/s41419-018-0443-y

**Published:** 2018-03-12

**Authors:** Qingzu Gao, Lihua Tang, Ling Wu, Kaitao Li, Hui Wang, Weidong Li, Juan Wu, Mingyi Li, Shuang Wang, Liang Zhao

**Affiliations:** 1 Department of Pathology, Nanfang Hospital, Southern Medical University, Guangzhou, China; 2 Department of Pathology, School of Basic Medical Sciences, Southern Medical University, Guangzhou, China; 3 Department of Pathology, The First Affiliated Hospital of Xinxiang Medical University, Xinxiang, China; 4 Department of Medical Oncology, Affiliated Tumor Hospital of Guangzhou Medical University, Guangzhou, China; 5 Radiotherapy Department, Affiliated Tumor Hospital of Guangzhou Medical University, Guangzhou, China

## Abstract

LIM and SH3 protein 1 (LASP1) enhances tumor growth and metastasis in various cancers, but its role in nasopharyngeal carcinoma (NPC) remains unclear. Herein, we investigated the role of LASP1 in NPC and explored the underlying mechanisms in NPC. Clinically, overexpression of LASP1 is associated with tumor metastasis and poor prognosis of NPC patients. Gain-of-function and loss-of-function assays showed that LASP1 promoted NPC cell proliferation, metastasis, and invasion in vitro and in vivo. Mechanistically, we observed clear co-localization between LASP1 and PTEN in NPC cells. LASP1 interacted with PTEN and decreased the expression of PTEN in NPC. The ubiquitination assay indicated that LASP1 overexpression increased PTEN ubiquitination. PTEN was known as a tumor suppressor by negatively regulating phosphoinositide 3-kinase/AKT signaling pathway. Rescue experiments showed that PTEN weakened LASP1-mediated cell proliferation, migration, and invasive abilities and decreased the phosphorylation of AKT in NPC cells. Our findings suggest that LASP1 has a crucial role in NPC progression via LASP1/PTEN/AKT axis, highlighting LASP1 as a therapeutic target for NPC.

## Introduction

Nasopharyngeal carcinoma (NPC) is a highly malignant tumor arising from the nasopharynx epithelium. Worldwide, there were an estimated 86 500 new cases of NPC, and 71% of the cases occurred in east and southeast parts of Asia^1, 2^. Genetic susceptibility and Epstein–Barr virus (EBV) is the main cause of NPC, and consistent elevation of EBV antibody titers is a well-established risk factor of progression of NPC. Although combining radiotherapy with chemotherapy was effective in controlling NPC without metastasis, the outcomes of patients with relapsed and distant metastasized NPCs remained poor^3, 4^. Therefore, there is an urgent need to uncover the underlying mechanism of NPC oncogenesis and metastasis, in the hope to find novel targets and provide new options for management of NPC.

The LIM and SH3 protein 1 (LASP1), a structural scaffolding protein and adhesion adaptor protein, is involved in many metastatic cancers. It is reported that LASP1 was overexpressed in 8–12% of human breast cancers and thought to be located in the cytoplasm exclusively^5^. *LASP1* gene is located at 17q12 in humans and encodes a protein containing an N-terminal LIM domain followed by two actin-binding sites and a C-terminal SRC homology SH3 domain^6^. Due to its composition that allows multiple protein–protein interaction, LASP1 is associated with various biological functions, including cell mobility, cell transduction, and cell morphology^7^. Although LASP1 has been reported to correlate with many malignant cancer, such as breast^6^, ovarian^8^, liver^9^, pancreatic^10^, colorectal^11^, esophageal carcinoma^12^, and medulloblastoma^13^, the relationship between LASP1 and NPC still remains elusive.

In the present study, we found that LASP1 was overexpressed in primary tissues of metastatic NPC compared to that of non-metastatic. High expression of LASP1 was associated with poor prognosis. Further we elucidated that LASP1 activated AKT signaling pathway by inhibiting the expression of PTEN. The ubiquitination assay showed that overexpression of LASP1 inhibited PTEN expression by increasing its ubiquitination. Our results suggested that LASP1 promoted NPC proliferation, metastasis, and invasion by regulating the phosphatase and tensin homolog/phosphoinositide 3-kinase/AKT (PTEN/PI3K/AKT) axis.

## Materials and methods

### Cell culture

A series of NPC cell lines (HNE1, HNE2, HNE3 5-8F, 6-10B, HONE1, CNE1, and C666) and the immortalized normal nasopharyngeal epithelial cell line (NP69) were a kind gift from the Laboratory of Otolaryngology-Head and Neck Cancer (Nanfang Hospital). The NPC cells were cultured in RPMI 1640 medium (Gibco, Grand Island, NY, USA) supplemented with 10% fetal bovine serum (NBCS Inc., Pasching, Austria). NP69 was maintained in keratinocyte/serum-free medium (Invitrogen, Carlsbad, CA, USA) with the growth factor supplements (Life Technologies, Gaithersburg, MD, USA). All of these cell lines were incubated in a humidified chamber with 5% CO_2_ at 37 ℃. For inhibitor treatment, 10 mmol/L PI3K inhibitor LY294002 (Cell Signal Technology, Danvers, MA, USA) was added in the cultured cells every 2 days.

### Tumor tissue samples

Two hundred and ten paraffin-embedded undifferentiated NPC specimens and 60 paraffin-embedded normal nasopharyngeal mucosa specimens were collected between 1994 and 1999 at the Department of Pathology, Southern Medical University. The study was approved by the Ethics Committee of Southern Medical University. The medical records of the patients were reviewed to collect the following clinicopathologic information: age, gender, clinical stage, and tumor node metastasis (TNM) stage. Survival data were available for a cohort of 210 patients. The median follow-up time was 43.7 months (range, 1.8–98.8 months). The pathological stage of all specimens was confirmed according to the 2016 NPC staging system of the World Health Organization.

### Animals

All animal experiments were carried out with the approval of the Southern Medical University Animal Care and Use Committee in accordance with the guidelines for the ethical treatment of animals. Nude nu/nu mice were maintained in a barrier facility in racks filtered with high-efficiency particulate air filter. The animals were fed with an autoclaved laboratory rodent diet. The mice in this study were purchased from the Experimental Animal Center of Southern Medical University, which is certified by the Guangdong Provincial Bureau of Science. All animal experiments involved ethical and humane treatment under a license from the Guangdong Provincial Bureau of Science.

### Western blot analysis

Protein expression was assessed by immunoblot analysis of cell lysates (20–40 μg) in radioimmunoprecipitation assay buffer in the presence of rabbit antibodies to β-tubulin, β-actin, glyceraldehyde 3-phosphate dehydrogenase (1:1000; Santa Cruz, California, USA); rabbit antibody to PTEN (1:1000; Proteintech); rabbit antibodies to p-AKT(Ser473), AKT, p-4-EBP1(Thr37/46), p-mTOR(Ser2448), mTOR (1:1000; CST, Danvers, MA, USA), and mouse antibody to LASP1 (1:1000; Millipore, USA). Immunosignal values of the protein were quantified by Gel-Pro analyzer. Relative protein abundance of phosphonate proteins was determined by normalizing to corresponding total protein levels. Relative protein abundance of total protein was determined by normalizing to corresponding endogenous control protein levels.

### Statistical analysis

Data were analyzed using SPSS version 19.0 software (SPSS, Chicago, IL, USA). Statistical significance of difference between groups was determined by a two-tailed paired Student’s *t* test. Significance of correlation between the expression of LASP1 and histopathological factors and the significance of the LASP1 expression between normal epithelial cell line and NPC were determined using Pearson’s *χ*^2^ test. Kaplan–Meier plots were performed to investigate the prognostic relevance of LASP1 in univariate analysis. Multivariate analysis was performed by applying Cox proportional hazards test. Statistical significance was established at *P* < 0.05.

## Results

### LASP1 expression is upregulated in NPC and associated with tumor progression and poor prognosis of patients with NPC

**Fig. 1:**
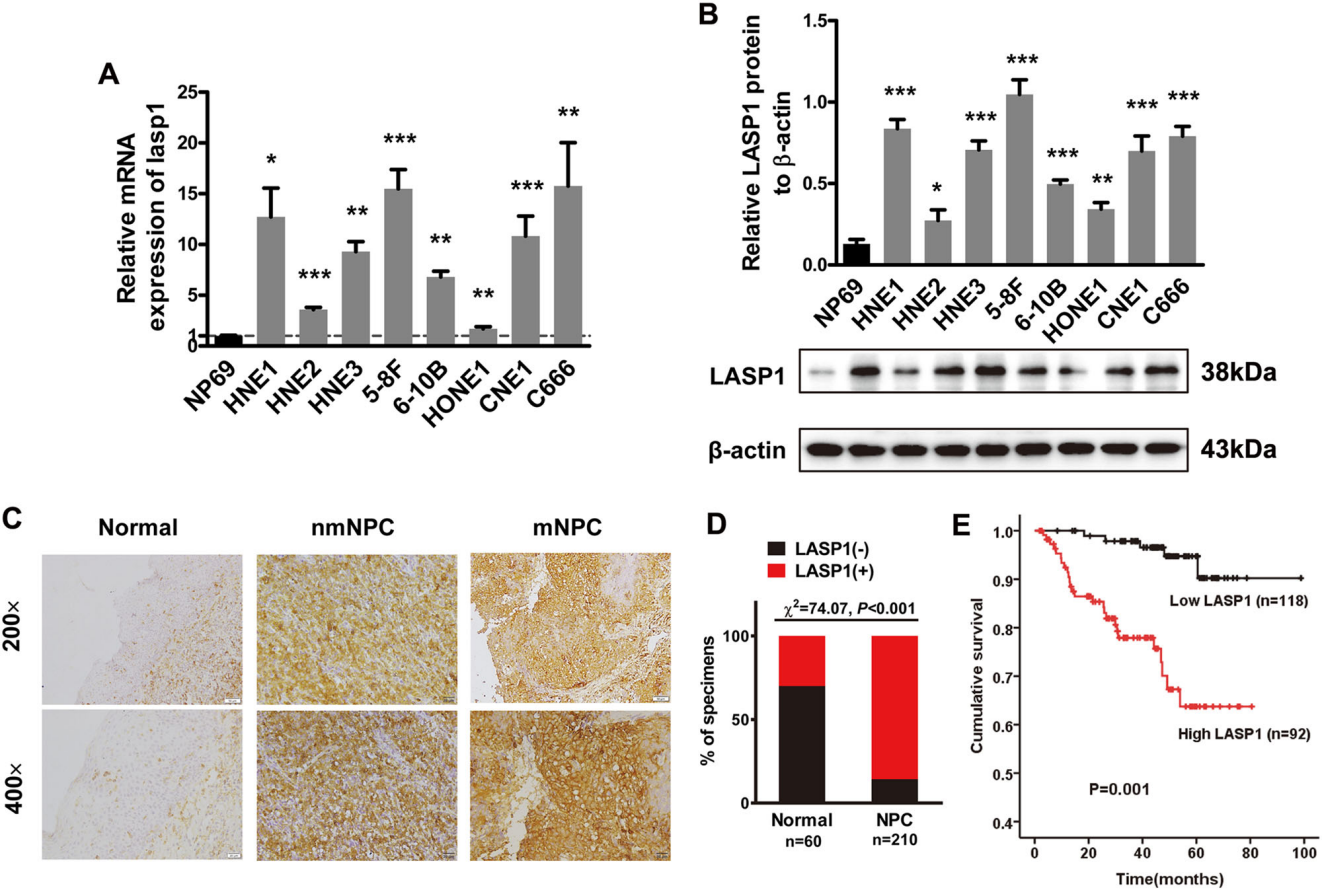
LASP1 expression is upregulated in metastatic NPC and associated with tumor progression and poor prognosis. **a** LASP1 expression assessed by real-time PCR in eight NPC cell lines and the normal epithelial cell line NP69. Date are means ± SD of three independent experiments (*P < 0.05, **P < 0.01, ***P < 0.001). **b** Western blot analysis for the expression of LASP1 in eight NPC cell lines and the normal epithelial cell line NP69. Immunosignal values were quantified by Gel-Pro analyzer. LASP1 expression in the individual cells was calculate as LASP1 expression relative to β-actin. Date are means ± SD of three independent experiments (*P < 0.05, **P < 0.01, ***P < 0.001). **c** Representative IHC analysis of LASP1 protein expression in normal nasopharyngeal squamous epitheliums, non-metastatic NPC (nm NPC), and metastatic NCP (mNPC) tissues. **d** Graphical illustration of statistical LASP1 expression distribution in 60 cases of normal tissues and 210 cases of NPC tissues. **e** Kaplan–Meier survival curves for CRC patients with distinct expression levels of LASP1

We detected the endogenous mRNA and protein expression of LASP1 in eight NPC cell lines and a normal nasopharynx epithelial cell line NP69. A relatively high expression of LASP1 was found in NPC cell lines than NP69. Of the NPC cell lines, HNE1, HNE3, 5-8F, CNE1, and C666 cells have high LASP1 expression than HNE2, 6-10B, and HONE1 cells (Fig. 1a, b). Among these cell lines, 5-8F and 6-10B were derived from the same NPC cell line SUNE-1. 5-8F has high metastatic potential, while 6-10B has low metastatic potential. Thus, we choose the two cell line for the following experiments. The immunohistochemistry (IHC) assay showed that only 30% of the normal nasopharynx epithelia were LASP1 (+) compared to 85.7% in NPC tissues (Fig. 1d). As compared to tumors without metastasis, LASP1 expression was stronger in metastatic tumors (Fig. 1c). To evaluate the clinical relevance of LASP1 expression, we analyzed its relationship with pathological features. As shown in Supplementary Table S1, LASP1 expression in NPC tissues was positively correlated with T classification (*P* = 0.015), N classification (*P* < 0.001), M classification (*P* = 0.002), and clinical stage (*P* < 0.001), but had no correlation with age and gender. Kaplan–Meier survival curves displayed a significant trend towards poorer survival for patients whose tumors showed high LASP1 expression, compared with those tumors that showed low LASP1 expression (Fig. 1d; *P* = 0.001).

### Knockdown of LASP1 in NPC cells inhibits cell proliferation, invasion, and migration, and enhances cell apoptosis

**Fig. 2:**
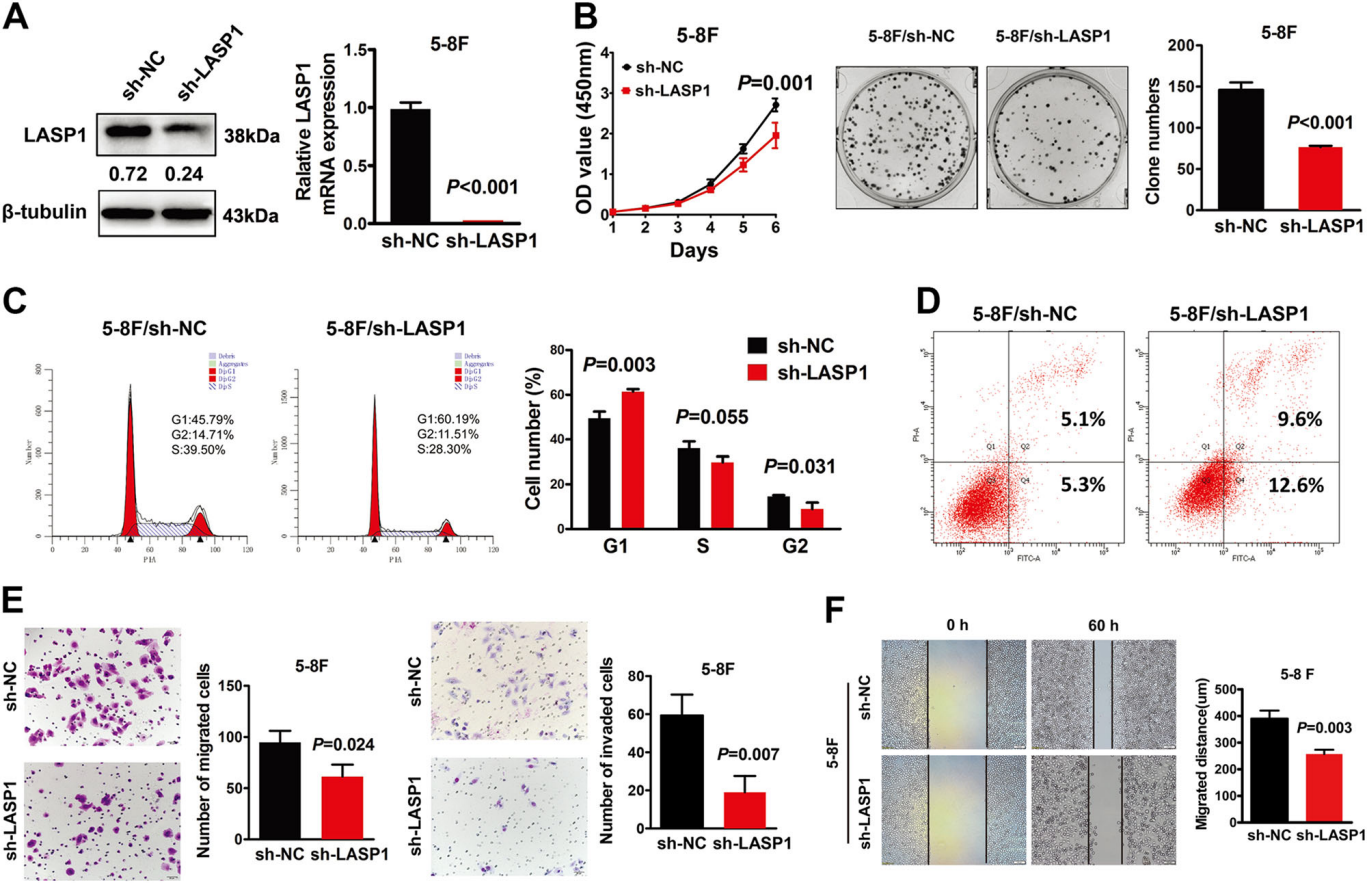
Knockdown of LASP1 in NPC cell inhibits cell proliferation, invasion, and metastasis, and enhances cell apoptosis. **a** Western blot and real-time PCR were performed to detect the protein and mRNA expression of LASP1 in 5-8F cells transfected with sh-LASP1 and sh-NC. The numbers under the band represent the relative immunosignal values of the protein. **b** Cell growth evaluated by the CCK-8 assay (left) and representative images of colony formation assay plates (right). The number of colonies containing >50 cells were counted. The error bars represent mean ± SD from three independent experiments. **c** The cell cycle phases of treated cells were evaluated by flow cytometry after being transfected with sh-LASP1 and sh-NC. The data are expressed as means ± SD (*n* = 3). **d** Flow cytometric analysis of apoptosis in vitro after treatment of cisplatin (100 μM) for 12 h. **e** Invasion and migration in 5-8F cells decreased after being transfected with sh-LASP1 in the transwell assay. Results are expressed as mean ± SD of three independent experiments. **f** Wound healing was delayed in shRNA-transduced cells compared to sh-NC cells after 48 h. Results are expressed as mean ± SD of migrated distance from three different points. Migrated distance = width (0 h) – width (48 h)

To investigate the effect of LASP1 silencing in NPC, we performed knockdown experiments in 5-8F cells, which exhibited relatively high expression of LASP1. Both the protein levels and the mRNA levels were markedly decreased in 5-8F cells after transfection with LASP1 short hairpin RNA (sh-LASP1), compared with negative control group (Fig. 2a). CCK-8 and colony formation assays revealed that knockdown of LASP1 markedly inhibited cell growth in 5-8F cells (Fig. 2b). The cell distribution was determined within the stage of the cell cycle by flow cytometry. Cells transfected with sh-LASP1 had a significantly higher proportion of cells in the G1/G0 phase (*P* = 0.003) and a significantly lower proportion of cells in the G2/M phase (*P* = 0.031), which indicated that sh-LASP1 inhibited cell proliferation by arresting the tumor cells at the G1/G0 phase (Fig. 2c). Next, we used flow cytometry to investigate the effect of LASP1 on apoptosis of NPC cells. The results showed that knocking down of LASP1 in 5-8F cells with sh-LASP1 increased the apoptosis cell numbers (Fig. 2d). The transwell assay and Matrigel-coated transwell assay indicated that knockdown of LASP1 compromised the migratory and invasive abilities of 5-8F cells, respectively (Fig. 2e). 5-8F cells displayed a significant reduction of cell motility after knockdown of LASP1 detected by the wound healing assay (Fig. 2f).

### Overexpression of LASP1 promotes NPC cell proliferation, invasion, migration, and tumorigenesis, and suppresses apoptosis

**Fig. 3:**
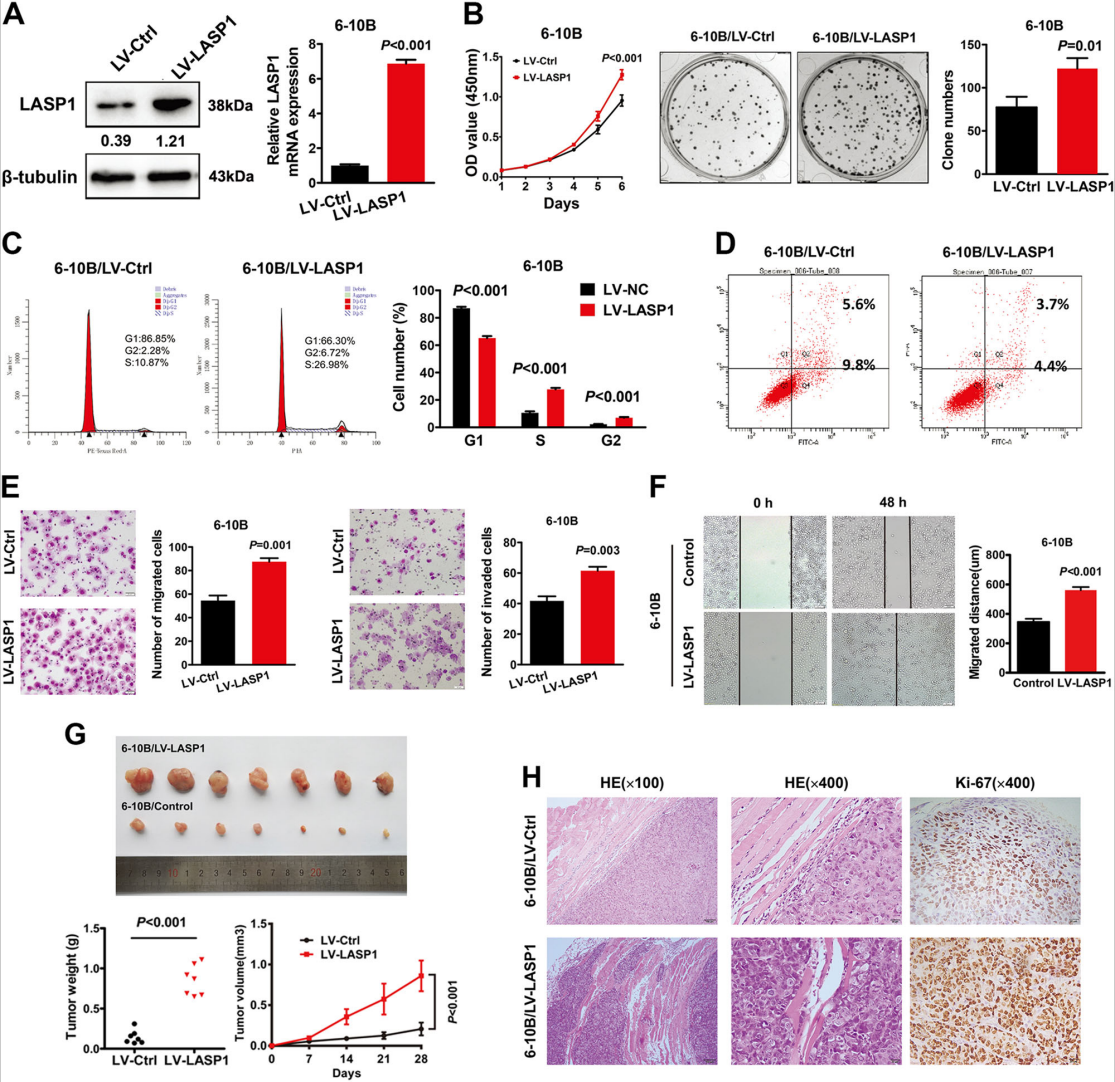
Overexpression of LASP1 promotes NPC cell proliferation, invasion, metastasis, and tumorigenesis, and inhibits cell apoptosis. **a** Western blot and real-time PCR were performed to detect the protein and mRNA expression of LASP1 in 6-10B-LASP1 cells and control cells. The numbers under the band represent the relative immunosignal values of the protein. **b** Cell growth ability was assessed by CCK-8 assays (left) and representative images of colony formation assay plates (right). The colonies containing >50 cells were counted. The error bars represent mean ± SD from three independent experiments. **c** The cell cycle phases of 6-10B were evaluated by flow cytometry after being treated with LV-LASP1 and LV-Control. The data are expressed as means ± SD (*n* = 3). **d** Flow cytometric analysis of apoptosis in vitro after treatment of cisplatin (100 μM) for 12 h. The number of apoptosis cell was reduced in 6-10B cells transfected with LV-LASP1 than control cells. **e** Migratory (left) and invasive (right) abilities of 6-10B cells was enhanced after being transfected with LV-LASP1 compared to control cells in the transwell assay. Results are expressed as mean ± SD of three independent experiments. **f** Wound healing ability was promoted in LV-LASP1 cells compared to control cells. Results are expressed as mean ± SD of migrated distance from three different points. Migrated distance = width (0 h)—width (48 h). **g** Resected xenograft tumors were (up) injected with indicated cells. The final tumor weight in the scatter plot graph (down left) and tumor growth curve over time (down right). **h** The representative HE and IHC images of subcutaneous tumors. LV-LASP1 tumors promoted skeletal muscle invasion and higher Ki-67 index than control tumors

To explore the influence of upregulation of LASP1 on NPC cells, we performed overexpression experiments in 6-10B cells, whose protein and mRNA levels were relatively low (Fig. 3a). Western blot and quantitative real-time PCR analysis showed that both protein and mRNA levels of LASP1 were enhanced in 6-10B cells after transfecting with lentivirus-mediated LASP1 vector (6-10B-LASP1). CCK-8 and colony formation assays indicated that overexpression of LASP1 increased cell growth in 6-10B cells (Fig. 3b). Then, we investigated the cycle profiles of 6-10B cells by flow cytometry. 6-10B-LASP1 cells displayed higher S phase (*P* < 0.001) and G2/M phase (*P* < 0.001) and lower G1/G0 phase (*P* < 0.001), which indicated that LASP1 enhanced cell proliferation (Fig. 3c). Compared to the control cells, LASP1-overexpressing 6-10B cells exhibited decreased apoptosis (Fig. 3d). We also observed that overexpression of LASP1 increased cell migration and invasion in the transwell assay and Matrigel-coated transwell assay. respectively (Fig. 3e). In wound healing assay, cell migration rate was remarkably increased in 6-10B-LASP1 cells as compared with the control cells (Fig. 3f).

In vivo, we used 6-10B-LASP1 cells and control cells to perform a tumorigenesis assay in nude mice. The tumors in the 6-10B-LASP1 group grew faster than those in the control group (*P* = 0.029). Tumor weights in the 6-10B-LASP1 group were markedly higher than those in the control group (Fig. 3g; *P* = 0.029). Moreover, tumors in the control group had a clear boundary with the surrounding regions. In contrast, the primary tumors that were derived from LASP1-overexpressing cells showed infiltrative growth, thereby invading the surrounding fat and muscle tissues (Fig. 3h). IHC confirmed that the tumors from the 6-10B-LASP1 group displayed much higher Ki-67 indices than tumors from the control group (Fig. 3h).

### LASP1 promotes NPC progression by activating PI3K/AKT-mTOR signaling pathway

**Fig. 4:**
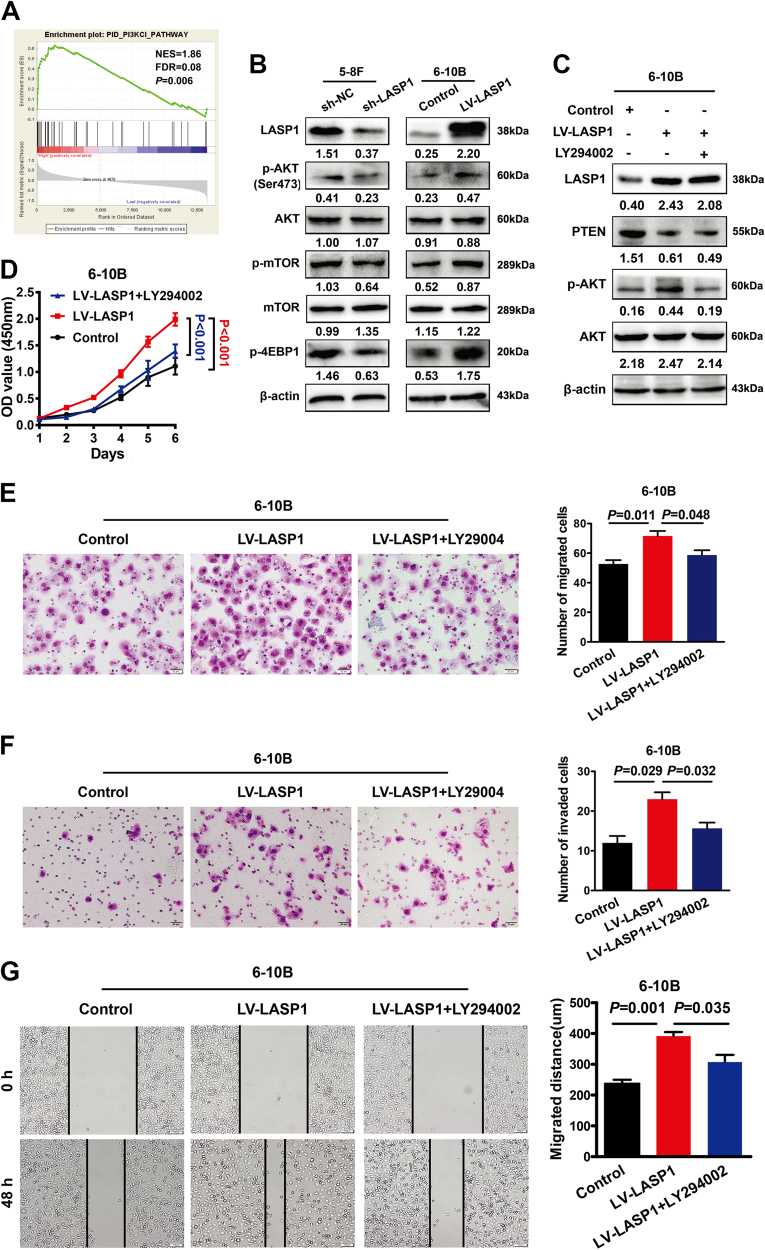
LASP1 promotes NPC progression by regulating PI3K/AKT signaling pathway. **a** GSEA demonstrated enrichment of the class I PI3K signal pathway in high LASP1 expression NPC group. **b** Western blot experiments were used to analyze the expression of relevant proteins in PI3K/AKT/mTOR signal pathway after LASP1 knockdown and overexpression. The numbers under the band represent the relative immunosignal values of the protein. **c** The upregulation of p-AKT induced by LASP1 via downregulation of PTEN were abrogated after administration of LY294002 in 6-10B cells. The numbers under the band represent the relative immunosignal values of the protein. **d** Cell proliferation promoted by LASP1 was counteracted after administration of LY294002. **e**,** f** Cell migratory and invasive abilities of 6-10B cells enhanced by LASP1 were counteracted after administration of LY294002. The data are expressed as means ± SD (*n* = 3). **g** Wound healing assay indicated that the cell motility of 6-10B cells enhanced by LASP1 was counteracted after administration of LY294002. The data are expressed as means ± SD (*n* = 3)

To explore the oncogenic signaling related to LASP1, we applied gene set enrichment analysis (GSEA) on the microarray data from the GSE 13597, which was downloaded from the GEO database of PubMed and it included microarray data from 25 patients with histologically confirmed undifferentiated NPC. The result indicated that PI3K class I signal pathway was enriched in the LASP1 overexpression group (Fig. 4a; NES = 1.86, false discovery rate = 0.08, *P* = 0.006). Knockdown of LASP1 by shRNA in 5-8F cells suppressed phosphorylation of AKT, mTOR, and 4EBP1, while overexpression of LASP1 in 6-10B upregulated the phosphorylation level of these proteins (Fig. 4b). Increased p-AKT by overexpression of LASP1 could be attenuated by LY294002 (an inhibitor of the PI3K/AKT signaling pathway) in 6-10B (Fig. 4c). The aggressive phenotypes of NPC cells promoted by LASP1 were also counteracted by LY294002 treatment (Fig. 4d–g).

### LASP1 negatively regulates PTEN by ubiquitin-mediated proteasome degradation

**Fig. 5:**
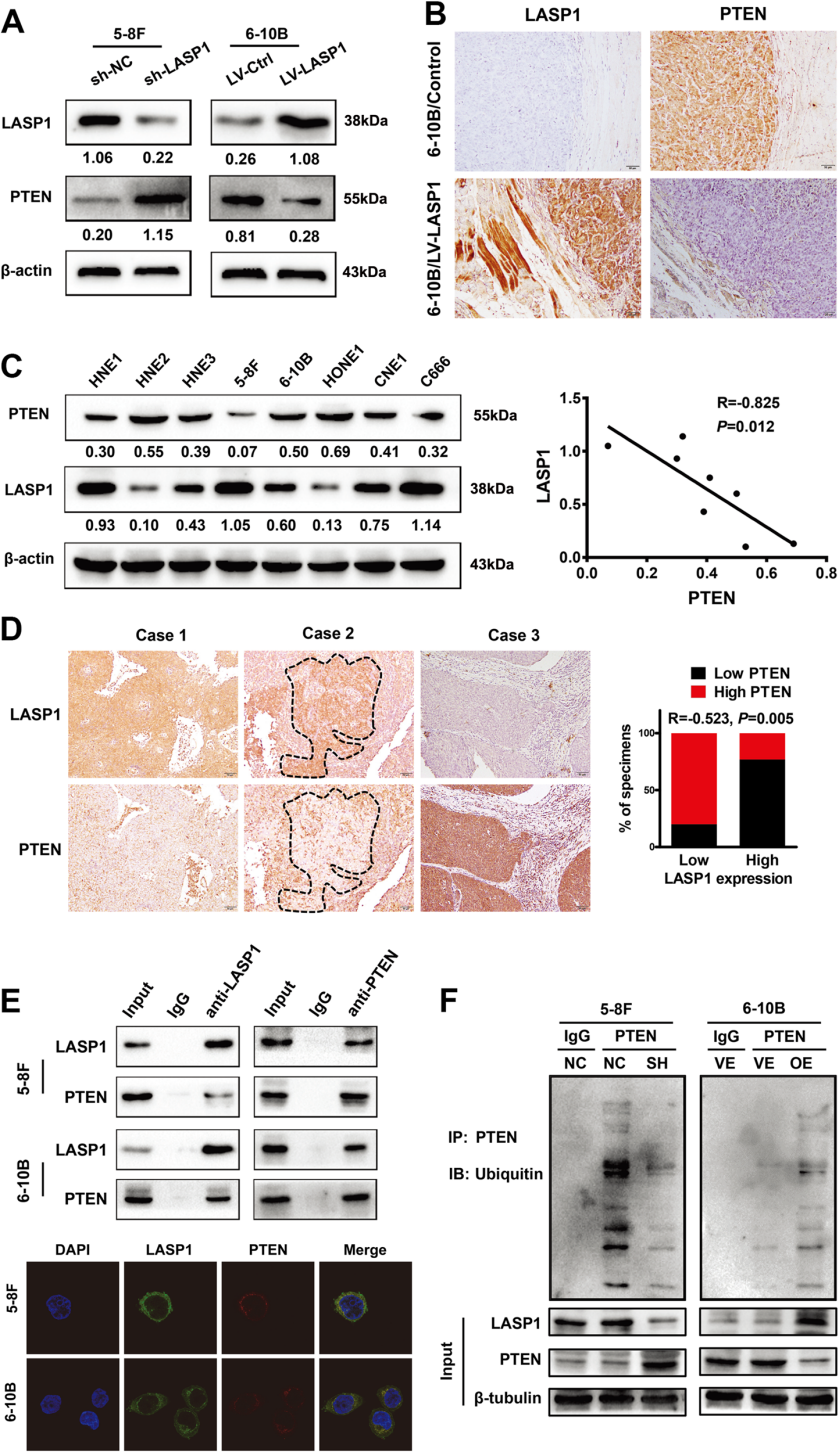
LASP1 negatively regulates PTEN in the protein level. **a** Western blot showed that the protein level of PTEN was increased in 5-8F cells after LASP1 knockdown by shRNA, while reduced in 6-10B-LASP1 cells compared to control cells. The numbers under the band represent the relative immunosignal values of the protein. **b** Representative images show IHC staining of LASP1 and PTEN in paraffin-embedded mice xenograft tumors. **c** Western blot analysis for the expression of PTEN and LASP1 in eight NPC cell lines. The numbers under the band represent the relative immunosignal values of the protein. The protein level of PTEN and LASP1 were negatively correlated. **d** IHC analysis indicated that PTEN expression was negatively correlated with LASP1 expression in NPC samples. **e** Endogenous protein interaction between PTEN and LASP1 in NPC cells (up). The cells were lysed and purified by anti-LASP1 or anti-PTEN affinity gel; protein pellets were analyzed by western blot with anti-LASP1 or anti-PTEN. The subcellular localization of PTEN and LASP1 in indicated cells was assessed by immunofluorescence staining (low). **f** Overexpressing LASP1 enhanced the ubiquitination of PTEN and LASP1 knockdown, which blocked the ubiquitination of PTEN

PTEN is a negative regulator of PI3K-AKT pathway. We speculated if LASP1 regulated PI3K-AKT via targeting PTEN. As shown in Fig. S1, LASP1 did not affect PTEN mRNA expression (Fig. S1). Western blot and Immunofluorescence (IF) results showed that LASP1 overexpression decreased the expression of PTEN and knockdown of LASP1 increased PTEN expression (Fig. 5a and S2). We detected the endogenous expression of LASP1 and PTEN protein in eight NPC cell lines and found a negative correlation between two proteins in these NPC cells (Fig. 5c; *R* = −0.826, *P* = 0.012). Similarly, IHC results from mice xenograft tumors and a cohort of 36 NPC tissues indicated that the protein level of PTEN was negatively correlated with LASP1 (Fig. 5b, d). Interestingly, co-localization of LASP1 and PTEN was observed by IF in NPC cells (Fig. 5e). We then examined whether LASP1 interacted with PTEN physically. Results of co-immunoprecipitated analyses showed that LASP1 bound to PTEN (Fig. 5e). Ubiquitination assay showed that overexpression of LASP1 in 6-10B cells led to increased ubiquitination of PTEN and decreased total protein level. An inverse result was seen in 5-8F (Fig. 5f).

### PTEN is essential for LASP1-mediated colorectal cancer cell aggressiveness

**Fig. 6:**
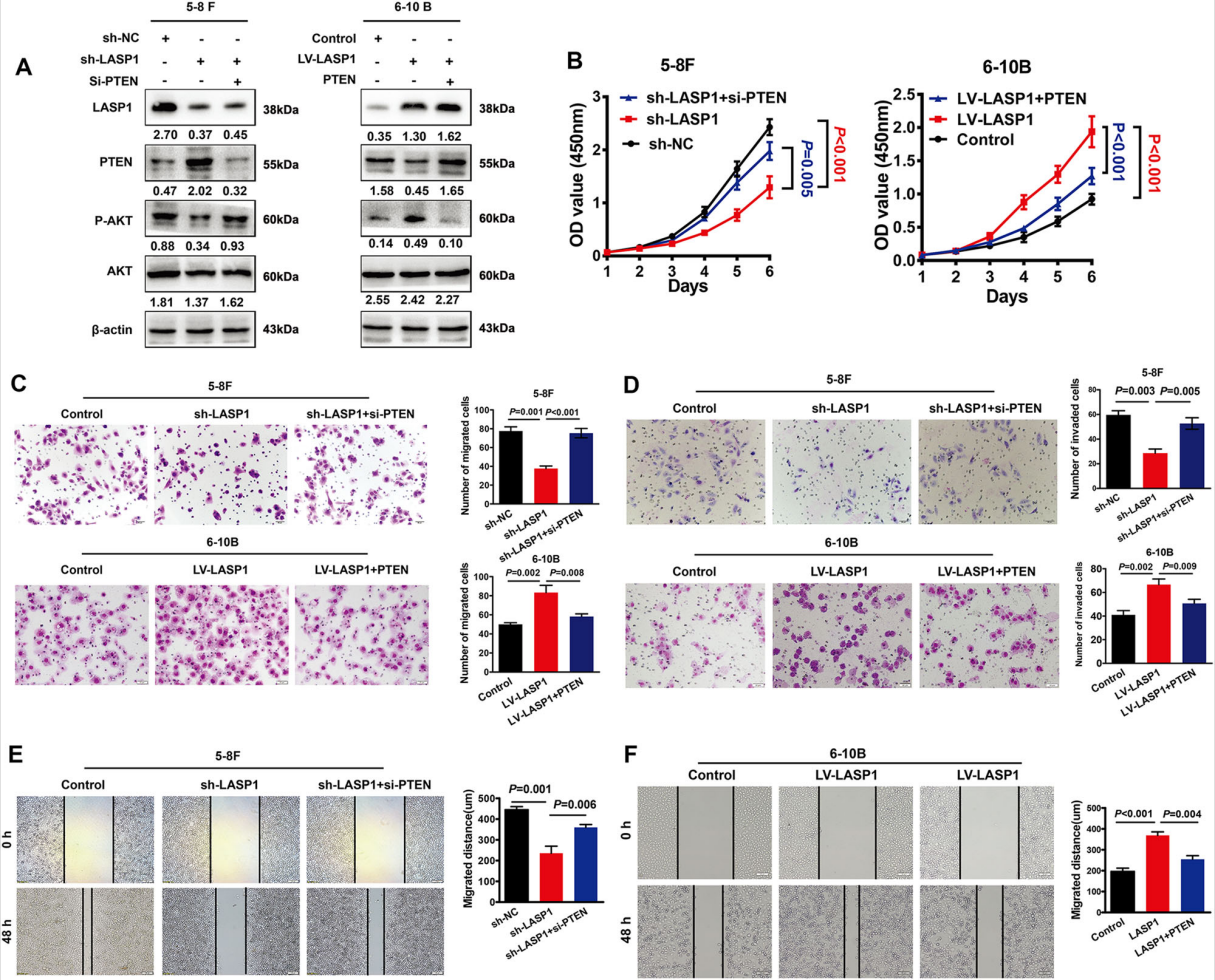
PTEN is essential for LASP1-mediated CRC cell aggressiveness. **a** PTEN upregulation and p-AKT downregulation induced by sh-LASP1 in 5-8F cells were neutralized by co-transfecting with si-PTEN. PTEN downregulation and p-AKT upregulation induced by transfecting LV-LASP1 in 6-10B cells were abrogated by co-transfecting with pcDNA3.1-PTEN. The numbers under the band represent the relative immunosignal values of the protein. **b-f** Cell growth ability was assessed by CCK-8 assays (**b**). Migratory (**c**) and invasive (**d**) abilities of indicated cells in the transwell assay. Motility ability of indicated cells was assessed by wound healing ability (**f**). Cell proliferative, migratory, and invasive abilities weakened by sh-LASP1 were restored after being transfected with pCDNA3.1-PTEN. Cell phenotypes promoted by LV-LASP1 were counteracted by co-transfecting with si-PTEN. Results are expressed as mean ± SD of three independent experiments

To investigate the role of PTEN in LASP1-induced cell aggressive phenotypes, we performed rescued experiments to observe the changes of PI3K/AKT signaling pathway and aggressive capacity of NPC cells. Results of western blot analysis indicate that the phosphorylated AKT level downregulated by silencing of LASP1 in 5-8F cells or upregulated by LASP1 overexpression in 6-10B cells were reversed by silencing or overexpressing LASP1 separately (Fig. 6a). Silencing PTEN by siRNA in LASP1-knockdown 5-8F cells rescued the proliferation, migration, and invasion abilities suppressed by silencing of LASP1, while restoration of PTEN in 6-10B-LASP1 cells attenuated proliferation, migration, and invasion abilities enhanced by LASP1 overexpression (Fig. 6b–f).

## Discussion

The *LASP1* gene was initially identified in a cDNA library of metastatic axillary lymph nodes from human breast cancer patients and strongly associated with lymph node metastasis and poor clinical prognosis^14^. The upregulation of LASP1 is observed in many malignant tumors, which suggests the oncological and clinical significance of LASP1^15^. Our study, for the first time, revealed that LASP1 contributed to proliferation, metastasis, and invasion of NPC. IHC staining showed that LASP1 is highly expressed in NPC tissues compared with that in adjacent normal squamous epithelium. Moreover, according to the clinicopathological data, high LASP1 expression was significantly associated with advanced TNM stage and shorter overall survival time of NPC patients. The positive correlation of LASP1 expression with the development of NPC indicated that the detection of LASP1 expression in resected NPC tissues could not only serve as a postoperative prognostic biomarker for NPC patients but also imply a potential therapeutic target for preventing NPC progression.

In this study, we observed a significant correlation between LASP1 overexpression and worse NPC clinical prognosis. However, multivariate analysis indicated that M classification and clinical stage, but not LASP1 expression, had positive predictive values for overall survival, suggesting that overexpression of LASP1 failed to serve as an independent prognostic factor for NPC. Therefore, more studies with a large sample size are required to verify these findings and establish the role of LASP1 in the prognosis of overall survival of patients with NPC.

GSEA on microarray data from the GEO database (GSE 13597) indicated that PI3K class I signal pathway was enriched in the LASP1-overexpressing NPC group. We confirmed that LASP1 activates PI3K/AKT in NPC cell lines. PI3K/AKT signaling pathway plays a crucial role in tumor progression^16^ and is constitutively activated in various cancers including lung cancer, gastric cancer, liver cancer, colorectal cancer, and NPC^17–21^. AKT resides in the cytosol in an inactive conformation and translocates to the plasma membrane when the cell is stimulated. The AKT PH domain has a high affinity for second messenger PI(3,4,5)P3 and binds to it preferentially over other phosphoinositides^22^. Therefore, activated PI3K is crucial for translocation of AKT to the membrane. Further, PTEN inactivates PI3K by converting PI(3,4,5)P3 into PI(4,5)P2, thus loss of PTEN function leads to over-activation of AKT^23^. Our study revealed the mechanism that LASP1 activated AKT, at least in part, by targeting PTEN and inhibiting PTEN expression. Nevertheless, LASP1 may mediate AKT phosphorylation via additional pathway. Thus, more experiments are needed to fully elucidate the mechanism underlying LASP1-mediated AKT activation.

PTEN can be regulated through multiple mechanisms, including transcriptional repression^24^, epigenetic silencing^25^, and microRNA regulation^26, 27^. Meanwhile, the stability of PTEN can be regulated by ubiquitination. It is reported that PTEN has at least two possible ubiquitination sites, lysine 289 and lysine 13^28, 29^, and NEDD4-1 has been identified as the major E3 ligase responsible for PTEN ubiquitination^30^. PTEN ubiquitination is also regulated by additional E3 ligases, including CHIP^31^, XIAP^32^ and WWP2^33^, and ret finger protein^34^. CHIP not only lead to PTEN ubiquitination but also ubiquitinates activated AKT. CHIP targets PTEN and activated AKT for degradation^31^, indicating the tight ubiquitination regulation occurring within the PI3K/AKT pathway. Our study attempted to investigate the mechanism by which LASP1 regulated PTEN expression. We found that LASP1 overexpression led to increased ubiquitination of PTEN and resulted in PTEN degradation. However, the exact mechanism as to how LASP1 induces ubiquitination of PTEN needs further study.

In summary, our study supports the oncogenic function of LASP1 in NPC. LASP1 is highly expressed in metastatic NPC tissues and closely associated with TNM stage of NPC patients. It promotes the cell proliferation, migration, and invasion in vitro and in vivo. LASP1 participates in the development and progression of NPC through LASP1/PTEN/PI3K/AKT axis. Our findings indicate that LASP1 may be a promising therapeutic target and serve as a prognostic biomarker of NPC.

## Electronic supplementary material

Supplementary files(DOC 695 kb)
