# Supplementary material for: LASP1 promotes nasopharyngeal carcinoma progression through negatively regulation of the tumor suppressor PTEN
Source: Cell Death Dis. 2018 Mar 12;9(3):393. doi: 10.1038/s41419-018-0443-y (PMC5847534; doi:10.1038/s41419-018-0443-y)
Supplement: Supplementary file 1 — Supplementary files(DOC 695 kb) [file 41419_2018_443_MOESM1_ESM.doc]

**Supplementary Materials and Methods**

**RNA isolation, reverse transcription, and quantitative real-time PCR**

Total RNA was extracted using Trizol reagent (Invitrogen). Total RNA was polyadenylated and underwent reverse transcription using PrimeScript™ RT Master Mix (TaKaRa, Dalian, China). Real-time PCR was carried out using a SYBR® Premix Ex TaqTM II (TaKaRa, Dalian, China) on an ABI 7500HT system. GAPDH was used as an endogenous control. All samples were normalized to internal controls, and fold changes were calculated through relative quantification (2-ΔΔCT). The primers used are shown in Supplementary Table S3.

**Immunofluorescence (IF)**

Cells were cultured on coverslips overnight, fixed with 4% paraformaldehyde for 20 min and treated with 0.5% Triton X-100 for 10 min. After blocking in 1% albumin bovine serum (BSA) at room temperature for 10 min, slides were incubated with rabit anti-PTEN (1:50; Proteintech) and mouse anti-LASP1 (1:200; Millipore) antibodies at 4°C overnight followed by washing with PBS three times. Coverslips were then incubated with fluorescein isothiocyanate- (FITC-) conjugated anti-rabbit IgG and tetramethylrhodamine- (TRITC-) conjugated anti-mouse IgG (1:200; Santa Cruz) for 60 min at room temperature, and then stained with 6-diamidino-2-phenylindole (DAPI; Invitrogen). The cells were observed under an Olympus FluoView™ FV1000 confocal microscope (Olympus, Hamburg, Germany). The images were taken and treated using OLYMPUS FLUOVIEW Ver.4.2a Viewer software.

**Cell migration and invasion analysis**

Cell migration was determined using 24-well transwell chambers with 8 μm pore size polycarbonate membranes (Corning Star; Cambridge, Mass,USA). 1 × 10^5^ cells were seeded on the top side of the membrane. The bottom chamber was prepared using 10% FBS as a chemoattractant. Cells were allowed to migrate through the porous membrane for 20 h at 37°C. The cells that stuck to the lower surface of the membrane were treated with a fixation/staining solution (0.1% crystal violet, 1% formalin, and 20% ethanol) for visualization. The cells were counted under a microscope in 5 randomly selected fields (original magnification, ×200). At least 4 chambers from 3 different experiments were analyzed.

Cell invasion was determined using 24-well transwell chambers with 8 μm pore size polycarbonate membranes (Corning Star; Cambridge, Mass, USA). 2 × 10^5^ cells were seeded on the top side of the membrane pre-coated with Matrigel (CORNING , Jiangsu, China) in RPMI1640 medium without serum. The incubation, fixation and quantification were performed as described.

**Cell proliferation assay and colony formation assay**

Cell proliferation assays were carried out using Cell Counting Kit 8 (CCK8) (Dojindo; Kumamoto, Japan). Cells were plated in 96-well plates at a density of 1 × 10^4^ cells per well and cultured in the growth medium. At the indicated time points, the number of cells in triplicate wells was measured at an absorbance at 450 nm of reduced WST-8 (2-(2-methoxy-4-nitrophenyl)-3-(4-nitrophenyl)-5-(2,4-disulfophenyl)-2H-tetrazolium monosodium salt). For the colony-formation assay, 300 viable cells were placed in each well in 6-well plates and maintained in a complete medium for 2 weeks. Colonies were fixed with methanol and stained with 0.1% (w/v) crystal violet.

**Wound healing assay**

Confluent monolayers of cells were maintained in serum-containing growth medium for at least 6 d and then in serum-free medium for 24 h. A 20μL plastic pipette tip was used to scratch the monolayers. The wounded cells were then cultured in a serum-free medium for an additional 48 h and photographed under an inverted phase contrast microscope. Three different points were marked on the plate, and the distance between each point and the edge of the scratch wound was measured before and after cell migration. The mean migration distance (μm) was calculated by subtracting the length after 48 h from that at 0 h. The result was expressed as a migration index, ie, the distance migrated by treated cells compared with the distance migrated by control cells. Experiments were repeated 3 times.

**Xenograft growth assay**

For the tumor growth assay, 1 × 10^6^ stable transfected cells were independently

injected subcutaneously into the left and right back of nude mice (n = 5/group). The tumor volume was calculated using the following formula: V = 0.5 × D × d^2^, where V represents volume, D represents the longitudinal diameter, and d represents the

latitudinal diameter. Mice were sacrificed 28 days after cell injection, and tumors were dissected and weighted.

**Cell cycle analysis**

Cells(1×10^6^ ) were collected, washed with phosphate buffer solution (PBS). The cells were resuspended in 1 ml DNA staining solution (MULTI SCIENCE; Hangzhou, China), then vortex the mix for 10 seconds. The mixture was then incubated at room temperature for 30 min in the dark. Analysis of cell cycle was carried out using flow cytometry.

**Cell apoptosis analysis**

Cells (5×10^5^) were washed with ice-cold PBS then resuspended in 500μL of binding buffer (KeyGen Biotechnology; Nanjing, China). Annexin V-FITC (5μL) (KeyGen) and propidium iodide (5μL) were added to the cell suspension. The mixture was then incubated at room temperature for 10min in the dark. Analysis of apoptosis was carried out using flow cytometry.

**Lentiviruses transfection assay**

Human LASP1 overexpression lentiviruses(LV-LASP1) and empty lentiviruses(LV-NC) were construct by GenePharma (Suzhou, China). For lentiviral transfection of 6-10B cells, 10ul LV- LASP1(6 ×10^8^TU/ml) or LV-NC(6 ×10^8^TU/ml) were added to a well containing 5x10^4^ cells, 1ml medium and 5 µg/ml polybrene. After 24 h of incubation, transfected cells were selected with 2 µg/ml puromycin (Sigma-Aldrich). Selected cells were maintained in growth medium with1 µg/ml puromycin.

**Vector construction and small interfering RNA (siRNA) synthesis and transfection**

The shortinterfering RNA (si-RNA) targed LASP1 (Gen Bank Accession No. NM_006148.3) was designed in the web <http://sirna.wi.mit.edu/> and was certified for LASP1 Specificity and none off-targe by BLAST. The corresponding cDNA sequence of si-RNA-LASP1 was chemically synthesised (Ruibio BioTech; Beijing, China) as complementary oligonucleotides. The synthetic oligonucleotide shRNA-LASP1: 5′- gatcccc GGTGAACTGTCTGGATAAG ttcaagaga CTTATCCAGACAGTTCACC ttttta-3′ and 5′- agcttaaaaa GGTGAACTGTCTGGATAAG tctcttgaa CTTATCCAGACAGTTCACC ggg-3′ was annealed and cloned downstream of HI promoter to construct recombinant pSUPER.puro/shRNA-LASP1 plasmid (sh-LASP1). The CDS sequence of PTEN (Gen Bank Accession No. NM_001304718.1) was PCR-amplified by TransStart FastPfu Fly DNA polymerase and subcloned into the Hind III and XBAI sites of pCDNA3.1 vector. The success of plasmid construction was verified by cDNA sequencing. Si-PTEN and si-NC was synthesized by GenePharma (supplementary table2). Cells were transfected with the plasmids, siRNAs using Lipofectamine 3000 (Invitrogen) according to the manufacturer's protocol.

**Co-immunoprecipitation assay**

Cells were harvested and lysed in RIPA with Protease and Phosphatase Inhibitor Cocktail (Sigma, USA). The protein was quantified using a BCA Protein Assay Kit (KeyGen, Biotechnology, China) and then diluted into 1 mg/ml with chilled wash buffer. Anti- LASP1((Millipore, USA,1:50) or PTEN(CST , USA,1:50) and anti-IgG (CST, USA, 1:50)antibody were added to the cells lysate and incubated overnight at 4°C. Protein A/G agarose beads were added into the protein mixtures and incubated overnight at 4°C with low-speed rotation. Afterwards, the protein mixture was washed three times with 1ml chilled PBS. All protein mixtures were diluted with wash buffer. After heat denaturation in 5% SDS-PAGE sample loading buffer, the protein samples were subjected to western blot assay.

**Ubiquitination assay**

Ubiquitination lelvel of PTEN was assessed in 5-8F cells stably transfected with sh-LASP1 vector or sh-NC and 6-10B cells stably transfected with LV-LASP1 or control empty lentivirus. The cells were lysed using RIPA buffer supplemented with PMSF and proteinase inhibitor. Immunoprecipitation was then performed using anti-PTEN(CST, USA,1:50) or anti-IgG, respectively. The immunoprecipitated proteins were tested by western blotting using anti-ubiquitin to evaluate the ubiquitination level. The inputs were subjected to western blot analysis with antibodies against LASP1, PTEN and β-tubulin, respectively.

**Immunohistochemistry (IHC)**

Dako Envision two-step method of immunohistochemistry was used according to the manufacturer’s instructions. The tumour sections (3um) were first deparaffinized and hydrated through graded alcohol to water. Then, antigen retrieval was performed by high-pressure heat in citrate buffer for 5 minutes. After natural cooling, the sections were treated with 3% H2O2 for 15 minutes to eliminate intrinsic peroxidase activity. Then the sections were incubated with primary antibodies overnight at 4℃. The primary antibodies include: mouse antibody to LASP1 (MAB8991; Millipore, Billerica, MA, USA, dilution 1:300, rabbit antibody to PTEN (22034-1AP , Proteintech, Wuhan, China dilution 1:300), and rabbit antibody to Ki-67 (27309-1-AP, Proteintech, Wuhan, China, dilution 1:1000). After washing with PBS for 3 times, the sections were incubated with the biotinylated secondary antibodies (dako, Denmark; Glostrup) for 60 minutes at room temperature. Sections were visualized with 3,3’-iaminobenzidine DAB and counterstained with hematoxylin for 30 seconds and then dehydrated and mounted in neutral gum.

To evaluation of the results, two pathologists scored all the sections independently. The final immunohistochemical score was combined the proportion of positive tumor cells and intensity of staining. The proportion score was evaluated as: 0, no positive tumor cells; 1,<30% tumor cells were stained; 2,30%-60% positive tumor cells; 3,>60% positive tumor cells. The intensity of staining was scored by the color of staining: 0, no staining; 1, light yellow; 2, yellow; 3, brown. The comprehensive IHC score was calculated by multiplying the proportion score by staining intensity score. Each section has a IHC score range of 0, 1, 2, 3, 4, 6 and 9. Cases with IHC score≤3 were defined as low expression and those with IHC score≥4 as high expression.

**Reference**

1. Niu, Y., et al., *LASP1-S100A11 axis promotes colorectal cancer aggressiveness by modulating TGFβ/Smad signaling.* Sci Rep, 2016. **6**: p. 26112.

2. Yang, Y., et al., *Epithelial-mesenchymal transition and cancer stem cell-like phenotype induced by Twist1 contribute to acquired resistance to irinotecan in colon cancer.* Int. J. Oncol., 2017. **51**(2): p. 515-524.

**Supplementary Figures**

**
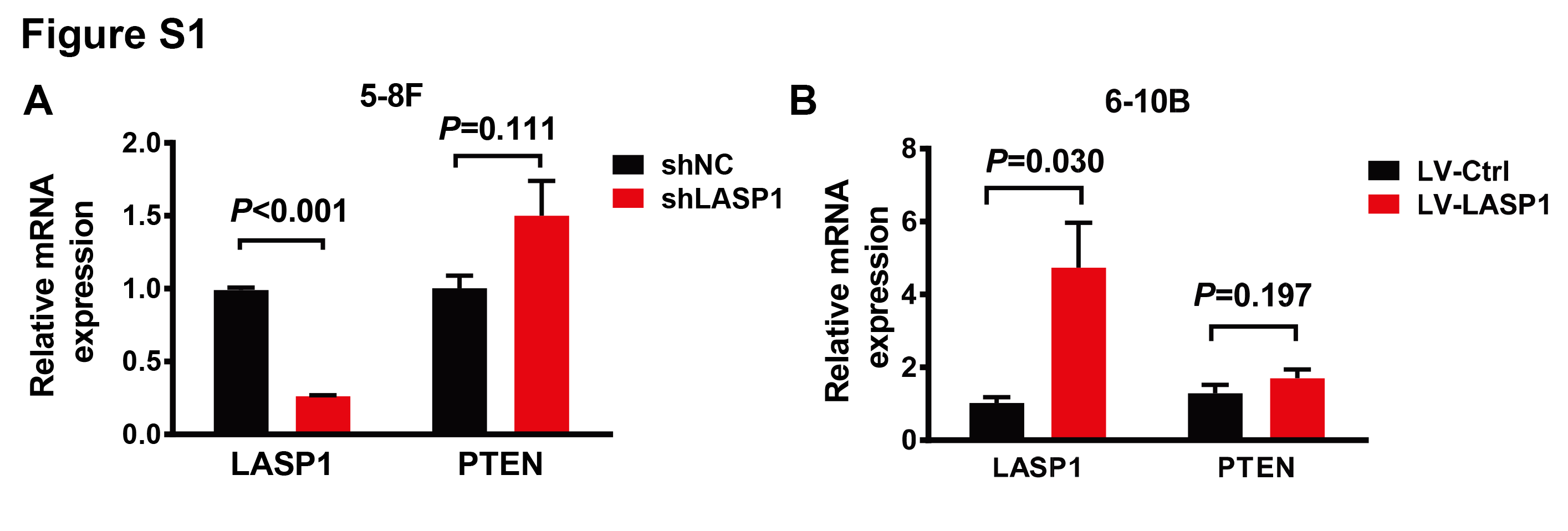
**

**Figure S1.**

**
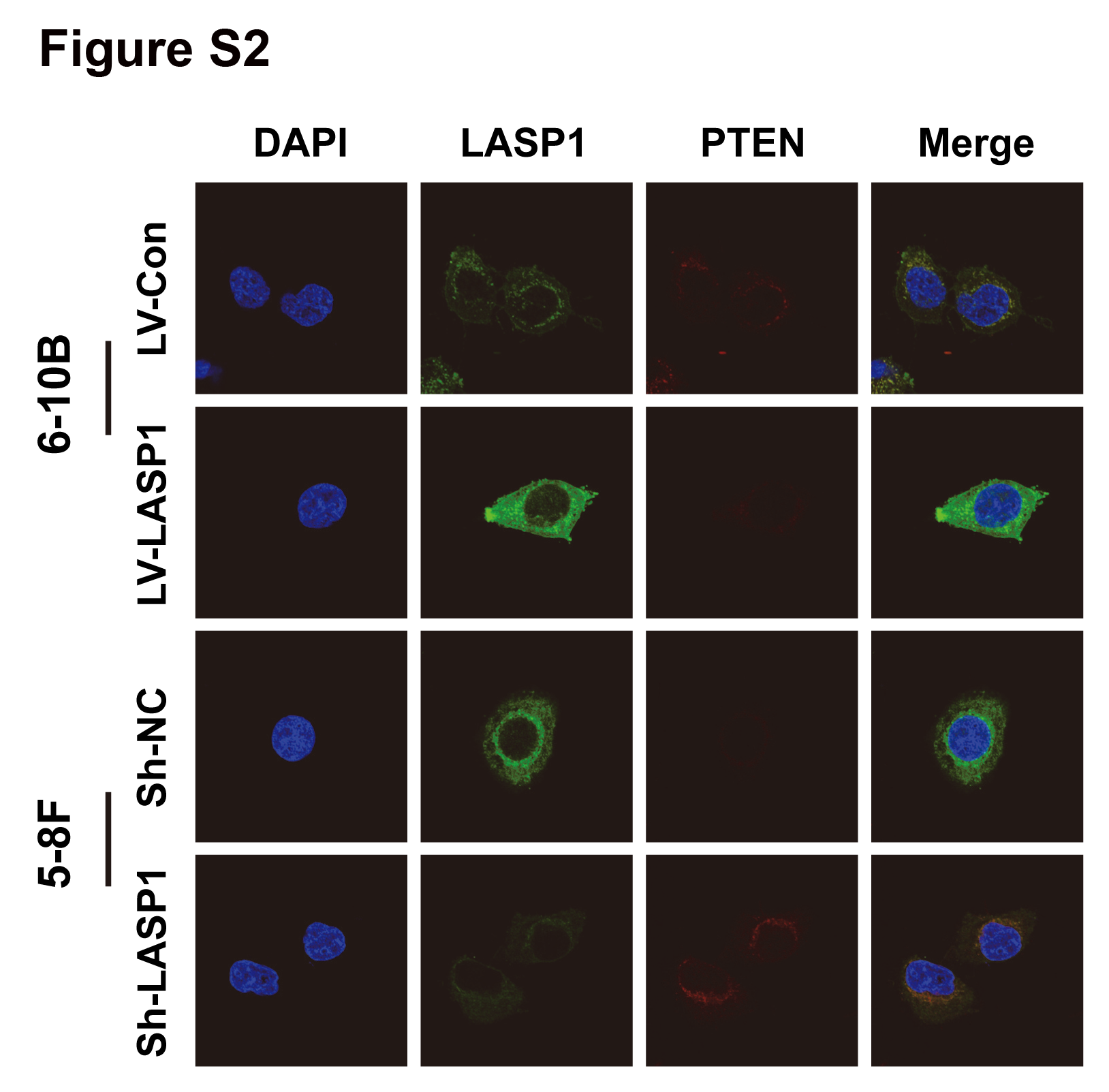
**

**Supplementary Table S1.** Correlation between the clinicopathological features and LASP1 expression

| **Characteristics** | | **Low LASP-1(%)** | **High LASP-1(%)** | **χ^2^ value** | ***p* value** |
| --- | --- | --- | --- | --- | --- |
| Gender | |  |  |  |  |
|  | Male | 82(50.9) | 79(49.1) | 1.027 | 0.331 |
|  | Female | 29(59.2) | 20(40.8) |  |  |
| Age(years) | |  |  |  |  |
|  | ≤ 47 | 59(54.1) | 50(45.9) | 0.147 | 0.782 |
|  | > 47 | 52(51.0) | 49(49.0) |  |  |
| T classification | |  |  |  |  |
|  | T1+T2 | 78(59.5) | 53(40.6) | 6.245 | 0.015* |
|  | T3+T4 | 33(41.8) | 46(58.2) |  |  |
| N classification | | |  |  |  |
|  | N0 | 100(61.7) | 62(38.3) | 22.384 | 0.000* |
|  | N1+N2+N3 | 11(22.9) | 37(77.1) |  |  |
| M classification | |  |  |  |  |
|  | M0 | 109(56.2) | 85(43.8) | 9.635 | 0.002* |
|  | M1 | 2(12.5) | 14(87.5) |  |  |
| Clinical stage | |  |  |  |  |
|  | I+II | 69(75.8) | 22(24.2) | 33.994 | 0.000* |
|  | III+IV | 42(35.3) | 77(64.7) |  |  |

* Statistically significant (P < 0.05).

**Supplementary Table S2.** Univariate and multivariate analyses of individual parameters for correlations with overall survival rate: Cox proportional hazards model

| **Variables** | **Univariate** | | |  | | **Multivariate** | | | | | |
| --- | --- | --- | --- | --- | --- | --- | --- | --- | --- | --- | --- |
|  | **OR** | **CI(95%)** | ***P* value** |  | | **OR** | | **CI(95%)** | | ***P* value** | |
| Age | 1.378 | 0.675-2.816 | 0.378 |  |  | |  | |  | |  |
| Gender | 0.456 | 0.159-1.305 | 0.143 |  | |  | |  | |  | |
| T stage | 2.730 | 1.324-5.626 | 0.007* |  | | 0.943 | | 0.420-2.118 | | 0.887 | |
| N stage | 1.835 | 0.901-3.738 | 0.094 |  | |  | |  | |  | |
| M stage | 0.044 | 0.018-0.108 | 0.000* |  | | 0.082 | | 0.031-0.216 | | 0.000* | |
| Clinical stage | 8.504 | 2.584-27.980 | 0.000* |  | | 5.787 | | 1.437-23.312 | | 0.014* | |
| LASP-1 expression | 3.296 | 1.545-7.032 | 0.002* |  | | 1.358 | | 0.579-3.185 | | 0.482* | |

Abbreviations: OR, Odds ratio; CI, Confidence interval.

* Statistically significant (P < 0.05).

**Supplementary Table S3.** RT-PCR primer sequences for human genes

| **Gene name** | **Forward primer** | **Reverse primer** | **Product length** |
| --- | --- | --- | --- |
| **LASP1** | CGAGAAGAAGCCCTACTGCAA | CTGCCACTACGCTGAAACCT | 167 |
| **PTEN** | TTTGAAGACCATAACCCACCAC | ATTACACCAGTTCGTCCCTTTC | 134 |
| **GAPDH** | AAGGTCGGAGTCAACGGATTTG | CCATGGGTGGAATCATATTGGAA | 159 |

**Supplementary Table S4.** siRNA sequences used for transfection

| **Gene name** | | **Sense 5’ to 3’** | | | **Antisense 5’ to 3’** |
| --- | --- | --- | --- | --- | --- |
| **PTEN** | GCUACAAGGAGGAGUUUGAUU | | | TCAAACTCCTCCTTGTAGC UU | |
| **Negative control (NC)** | UCUCCGAACGUGUCACGUTT | | ACGUGACACGUUCGGAGAATT | | |
